# Effective Attributes Quantification to Bridge Gap between Elastic Properties and Reservoir Parameters in Self-Resource Rocks

**DOI:** 10.1038/s41598-020-59311-w

**Published:** 2020-02-13

**Authors:** Muhammad Abid, Jianhua Geng

**Affiliations:** 1 State Key Laboratory of Marine Geology, Tongji University, Shanghai, China; 2 Center for Marine Research, Tongji University, Shanghai, China; 3 School of Ocean and Earth Science, Tongji University, Shanghai, China

**Keywords:** Geophysics, Crude oil

## Abstract

The successful production of unconventional resources such as shale gas is highly dependent on its two reservoir properties, organic matter and rock brittleness. High resolution spatially characterization of these two unconventional reservoir properties needs surface reflection seismic data. However, to delineate these two parameters on seismic scale is a challenging task because poor correlation is observed between these parameters and elastic properties of the rock. To encounter this adversity in current study we proposed effective attributes method in which organic shale reservoir properties are divided into their hard and soft elastic response. From the analysis of worldwide laboratory dataset, we find that hard and soft components have shown us much better linear correlation with P- and S- wave impedance. The proposed effective attributes, helped us to reduce the gap between unconventional reservoir properties and seismic characteristics. These attributes are the main controlling factor for rock elastic properties and exhibit information about hydrocarbon generation capacity and rock brittleness. A well data example from Sembar shale has also shown successful results for proposed effective attributes methodology. These attributes application on inverted P-wave impedance seismic data of employed organic shale reservoir have shown productive results to quantify its unconventional prospect on seismic scale. The approach used in this study can be confidently employed to assess unconventional reservoir potential in other parts of the world.

## Introduction

Organic shale has been habitually regarded as the source rock in conventional petroleum system. Recently by application of horizontal drilling along with multistage hydraulic fracturing have expand the ability to get production from these low permeability self-resource reservoirs. Now unconventional resources have become an important source of energy worldwide^1^. The successful production of these self-resourcing rocks largely depended on amount of organic matter (OM) a soft component and shale brittleness a hard component. The organic matter decides about the potential of rock for generating oil and gas resources^2^. The brittleness quantification is from the point of view of reservoir drilling and is known as brittleness index (BI)^3,4^.

Generally, well logs are used to quantify organic matter and to do so several methods have been proposed by different researchers^5–7^. The brittleness index can be inferred through elastic properties or minerals content of the rock^8^. In conventional petroleum system most of the focus was on reservoir rock so, there are very limited number of wells which target source rock. The substitute is to get source rock information from surface reflection seismic data which will also cover the regional views and would be better way to reveal hydrocarbon potential basin wide.

**Figure 1:**
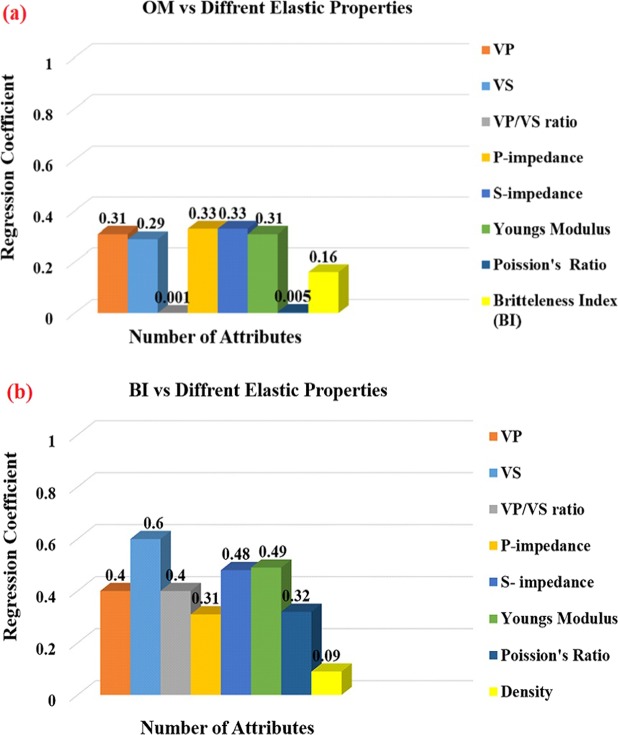
Histogram showing the values of correlation between different elastic properties, (**a**) Organic matter and (**b**) Brittleness Index (Laboratory dataset of world-famous organic shale reservoirs).

Several researchers have analyzed seismic response of source rock as function of its different properties. Vernik and others have evaluated organic matter, velocity and anisotropy relationship in source rocks^9–11^. Løseth *et al*.^12^ link acoustic impedance with organic matter to delineate it on seismic data. Per Avseth and Carcione^13^ developed rock physics models for screening organic richness. Zhao *et al*.^1^ use sum of organic matter and porosity is to delineate organic shale to identify reservoir parameters. Del Monte *et al*.^14^ used seismic inversion and rock physics modeling to estimate organic matter on seismic data. All previously developed methods cannot be used widely because restricted to particular area or formation on which these were investigated. For example, acoustic impedance method^12^ shows poor correlation with organic matter in most of organic shale formations (Fig. 1).

Still one of the main challenge for geophysicists is to remotely locate unconventional resources on seismic data because there exists a gap to directly link elastic parameters and organic shales reservoir properties. This gap exists due to heterogeneous nature of shales, mineralogical composition and hydrocarbon maturity level^1,10,15–19^. These all factors lead to a poor correlation between organic shales reservoir properties and rock elastic properties (Fig. 1).

Generally, the organic matter has impact to soften the elastic response while brittleness has opposite impact. We propose a concept to separately link the soft and hard components of organic shales with elastic properties to directly to characterize it on seismic scale. This approach is favorable to simplify complex factors and can help to relate relevant elastic properties to reservoir parameters.

The main objective of current study is to evaluate effective attributes to bridge the gap between reservoir parameters and their seismic characteristics in self-resource rocks. We propose this attributes method on worldwide laboratory datasets of organic shales through which unconventional sweet spots can be easily quantified on seismic scale. These attributes have shown much better correlation with P-and S-wave impedance of the rock.

The structure of the paper is organized as follows: Initially we introduce the dataset available for this this study. In coming part, we describe proposed methodology. Then, we have results and discussions. Finally, in last part conclusions are presented.

## Available Data

**Figure 2:**
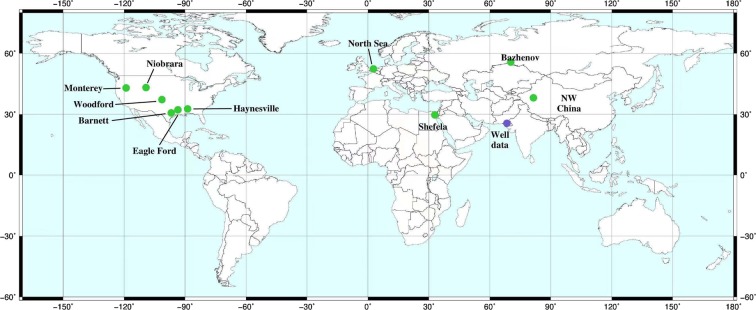
Approximate locations of shale formations used in this study (green dots). The blue dot represents well-log data.

We have two datasets the first laboratory based dataset is obtained from literature^1,10,11,17,20,21^. The laboratory dataset of organic shales includes organic matter content, density, and ultrasonic velocities. We have utilized 87 core samples attained from Monterey Formation, Eagle Ford Formation, Northwestern China oil shale, Bazhenov Formation, Shefela shale, Woodford Formation, Niobrara Formation, Haynesville Formation, Barnett Formation and North Sea shale. The lithology of these samples varies from fine siliceous shales to carbonate rich shales^1^. The organic matter content is in a range from less than 1 to 36% (volume fraction). The locations of shale reservoirs used in this study is shown in Fig. 2.

The second dataset include one logging data of well Khajari-01 along with its formation tops and geochemical data. The target formation in this well is Sembar shale from Lower Indus basin Pakistan. We also attained one prestack seismic line to show real data seismic application.

## Method

Brittleness is a critical indicator for hydraulic fracturing candidate screening in unconventional reservoirs. It is more practical to estimate brittleness on the basis of mechanical properties of the rock^22,23^. The brittleness evaluation parameters such as Young’s modulus, Poisson’s ratio and Lame’s parameters can be directly quantified through extracted rock elastic properties.

Organic matter in rock defines its hydrocarbon generation potential. The rock having high organic matter content considered as good candidate for production.

Direct information that can be reliably extracted from seismic data through seismic inversion is about its elastic properties such as P- and S-wave velocity, density or P- and S-wave impedance^24^. But these elastic properties are not precisely sensitive to organic shale reservoir properties and often shows poor correlation.

Initially we calculated different rock geomechanical parameters such as Young’s modulus, Poisson’s ratio, Lame parameter and shearing modulus by using ultrasonic velocities and densities of the available dataset. Young’s modulus and Poisson’s ratio are calculated by using following equations.

1 $${E}={{\rho }}_{{b}}\frac{(4-3\Delta {{t}}_{{S}}^{2}/\Delta {{t}}_{{C}}^{2})}{\Delta {{t}}_{{S}}^{2}(1-\Delta {{t}}_{{S}}^{2}/\Delta {{t}}_{{C}}^{2})}$$

2 $$\nu =\frac{2-\varDelta {t}_{S}^{2}/\varDelta {t}_{C}^{2}}{2(1-\varDelta {t}_{S}^{2}/\varDelta {t}_{C}^{2})}$$

Where E represents Young’s modulus, *ν* is Poisson’s ratio, ${{\rho }}_{{b}}$ is bulk density, the ${\Delta }{{t}}_{{C}}$ and ${\Delta }{{t}}_{{S}}$ are compressive and shear slowness.

The areas with high Young’s modulus and low Poisson’s ratio are more productive for hydraulic fracking due to their high brittleness^3^. The λρ and μρ is another widely-used geophysical method to predict rock brittleness, the zones with small λρ and high μρ are considered as brittle zones^25,26^ where λ, μ and ρ are Lame coefficient, shearing modulus and density respectively.

From the inversion of Pre- stack seismic data, all these parameters can be evaluated directly but to get successful production organic matter is equally important. These parameters are not directly sensitive to organic matter. The direct correlation these parameters with organic matter results in low regression coefficient. This low regression is meaningless to evaluate reservoir potential.

We proposed various effective attributes by integration of different strong (brittleness) and weak components (organic matter) to their respective P-and S-wave impedance. Similarly, Guo *et al*.^27^ divide Young’s modulus by Poisson’s ratio to define brittleness index. The organic matter and Poisson’s ratio both have similar response to softening the rock impedance, while Young’s modulus have opposite effect. If we divide Young’s modulus by sum the organic matter and Poisson’s ratio it can provide useful source rock information with much better correlation to rock impedance. We define the first effective attribute EA_1_:

3 $${{\rm{EA}}}_{1}=\frac{{E}}{{OM}+{\nu }}$$

Where OM represents organic matter in volume fraction.

The same effect on rock impedance is observed for the case of μρ as for observed in case of Young’s modulus. The second effective EA_2_ attribute is defined as follows

4 $${{\rm{EA}}}_{2}=\frac{{\mu }{\rho }}{{OM}+{\nu }}$$

In next part we calculated elastic brittleness. One method used to calculate brittleness from mechanical parameters, include Poisson’s ratio and Young’s modulus^28^.

5 $$\text{BI}\,=\,\frac{\frac{(E-{E}_{\min })}{({E}_{\max }-{E}_{\min })}+\frac{(\nu -{\nu }_{\max })}{({\nu }_{\min }-{\nu }_{\max })}}{2}$$

In Eq. 5, BI stands for elastic brittleness, *E*_max_ and *E*_min_ represent maximum and minimum Young’s modulus. Similarly, the ${{\nu }}_{{\max }}$ and ${{\nu }}_{{\min }}$ are maximum and minimum Poisson’s ratio. The brittleness has positive impact on elastic impedance of the rock, so we divided it by soft components the sum organic matter and Poisson’s ratio. We define the third effective attribute EA_3_ as follows:

6 $${{\rm{EA}}}_{3}=\frac{BI}{OM+\nu }$$

For conventional logging data initially we calculated the TOC in well through ∆logR method^5^. The TOC was converted to organic matter by equation proposed by^29^.

In coming part, these three formulated attributes were correlated with P- and S-wave impedance of worldwide laboratory datasets of different basins. Finally, all previously described elastic parameters and reservoir properties were integrated to evaluate effective attributes on available well data of Khajari-01.

## Results

### Laboratory data example results

**Figure 3:**
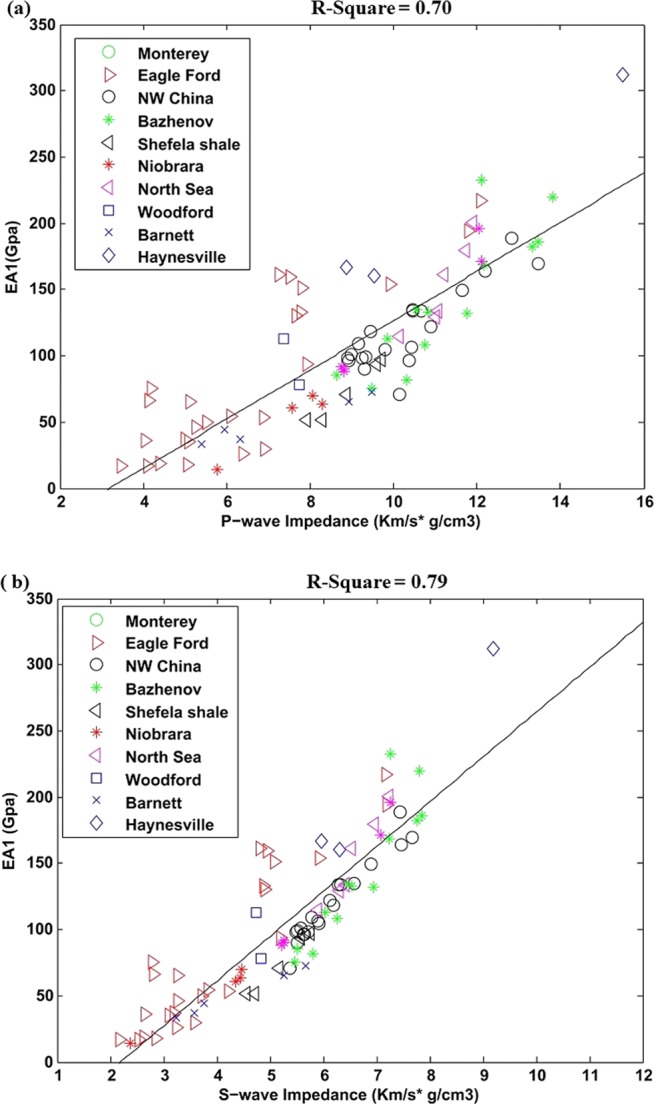
(**a**) Correlation between P-wave impedance and EA_1_, (**b**) Correlation between S-wave impedance and EA_1_.

In order to verify the accuracy of proposed method initially we used laboratory dataset of various organic shale reservoirs worldwide. The calculated effective attributes EA_1_ correlation with rock P- and S-wave impedance is shown in Fig. 3. The different color bars are representing different datasets from various organic shales reservoirs. The square of linear regression coefficient R^2^ in the case P impedance is 0.70 while, for S impedance is 0.79. The linear equations obtained through this correlation are given below in Eq. 7a and 7b. The constants in this equation can vary with varying datasets.

7a $${{\rm{EA}}}_{1}=18.56{I}_{P}-59.14$$

7b $${{\rm{EA}}}_{1}=33.83{I}_{S}-74.15$$

In Eq. 7a and 7b, *I*_P_ and *I*_S_ represents P- and S-wave impedance respectively.

**Figure 4:**
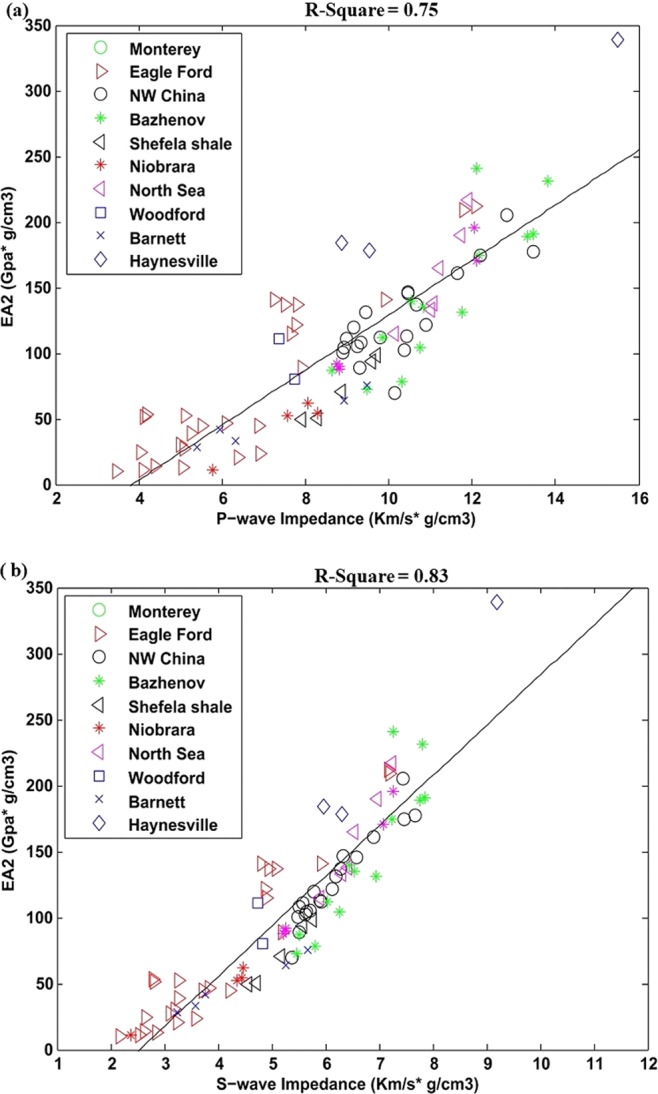
(**a**) P-wave impedance correlation with EA_2_, (**b**) S-wave impedance correlation with EA_2_.

The second effective attribute EA_2_ correlation with P- and S-wave impedance is shown in Fig. 4. The S- impedance have shown slightly better correlation as compared to P impedance. The square of linear regression coefficients R^2^ is 0.83 and 0.75 respectively. The linear equations obtained by this correlation are mentioned in Eq. 8a and 8b.

8a $${{\rm{EA}}}_{2}=20.96{I}_{P}-80.51$$

8b $${{\rm{EA}}}_{2}=38.01{I}_{S}-96.44$$

**Figure 5:**
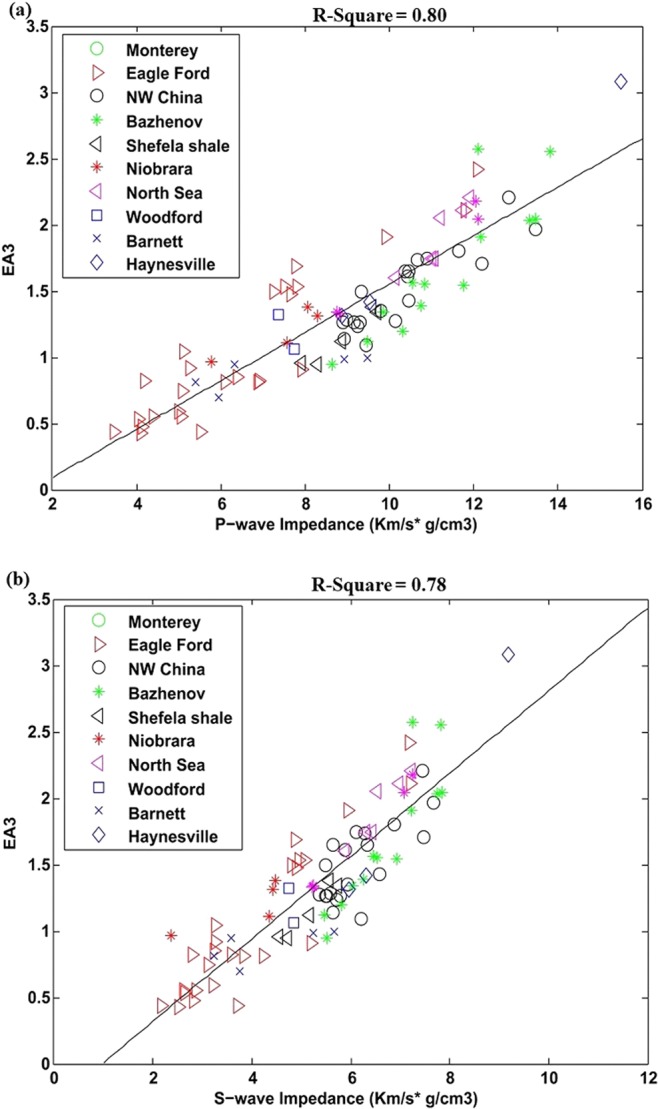
**(a)** EA_3_ correlation with P-wave impedance, **(b)** EA_3_ correlation with S-wave impedance.

Figure 5 shows EA_3_ on laboratory dataset and its correlation with P- and S-wave impedance. The square of liner regression coefficient R^2^ is about 0.80 and 0.78. Linear equations through the correlation are given in Eq. 9a and 9b.

9a $${{\rm{EA}}}_{3}=0.18{I}_{P}-0.27$$

9b $${{\rm{EA}}}_{3}=0.31{I}_{S}-0.30$$

### Well data example

**Figure 6:**
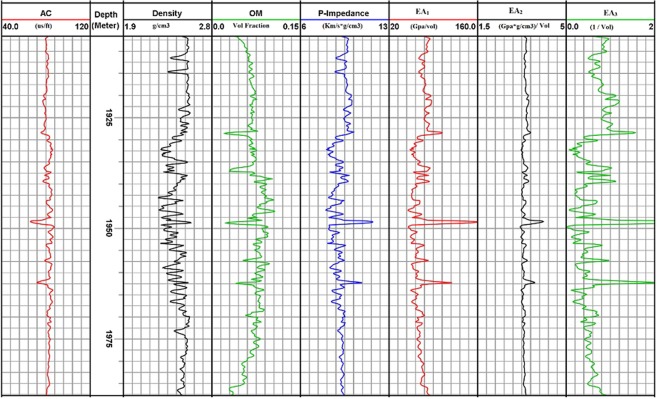
Calculation results for sonic log, density log, organic matter, P-wave impedance and three effective attributes across depth in well Khajari-01.

In well Khajari-01, sonic and density log along with its calculated organic matter across depth are displayed in the first four tracks respectively (Fig. 6). Track 5 represent P- wave impedance while next tracks have calculated three effective attributes EA_1_, EA_2_ and EA_3_. From Fig. 6 it is clear that attributes follow *I*_P_ closely, as *I*_P_ increase there is also increase in these attributes.

**Figure 7:**
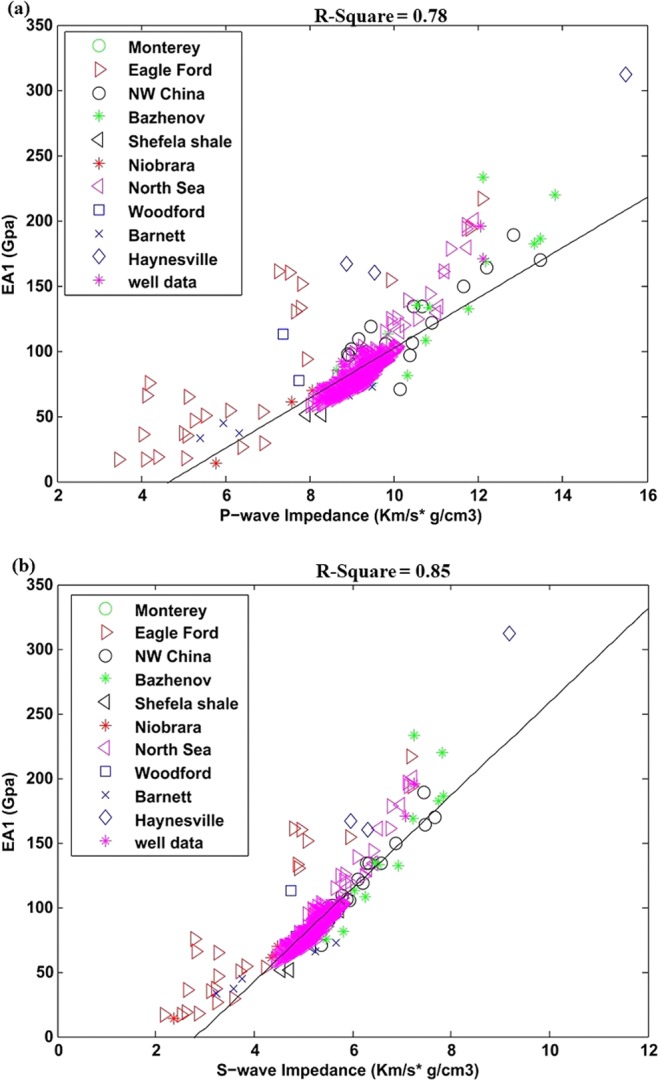
(**a**) Correlation between P-wave impedance and EA_1_ for well data with overlaid laboratory data, (**b**) Correlation between S-wave impedance and EA_1_ for well data with overlaid laboratory data.

The calculated effective attribute EA_1_ and its cross-plots with P-and S-wave impedance in well Khajari-01 with overlaid laboratory data is shown in Fig. 7. Most of the points from both datasets are aligned and showing same trend. The linear regression is generated just across well data which is relatively higher for the case of S-wave impedance. The square of linear regression coefficient R^2^ for P-impedance and S-impedance are 0.78 and 0.85 respectively. The linear equations of EA_1_ obtained from regression analysis of well data are given below.

10a $${{\rm{EA}}}_{1}=25.75{I}_{P}-154$$

10b $${{\rm{EA}}}_{1}=38.94{I}_{S}-119.18$$

**Figure 8:**
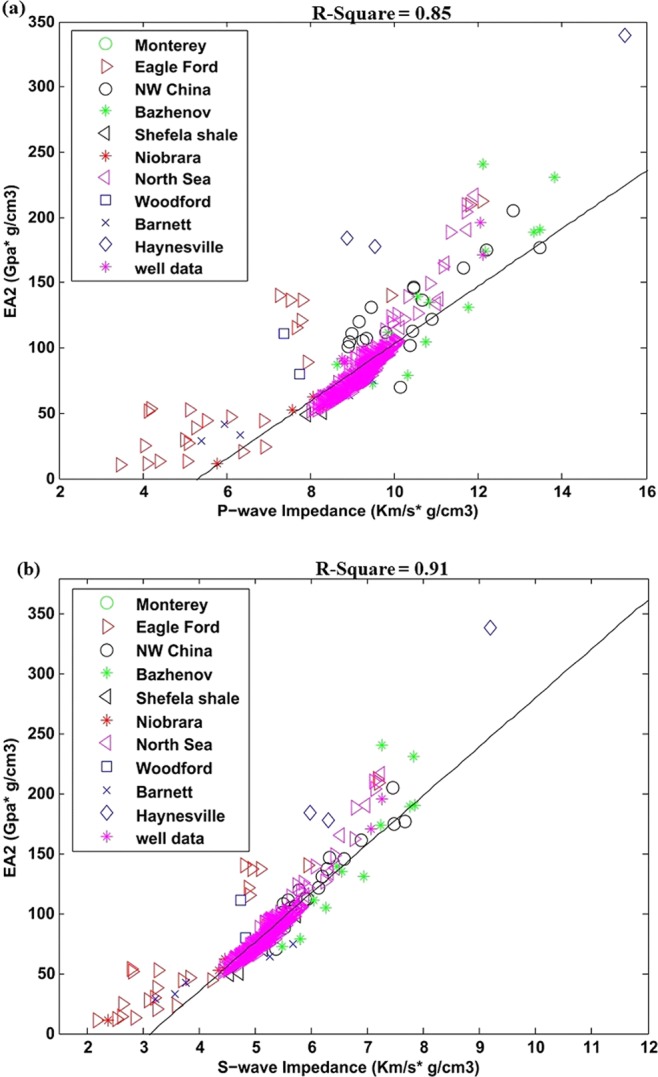
(**a**) P – wave impedance correlation with EA_2_ for well data with overlaid laboratory data, (**b**) S-wave impedance correlation with EA_2_ for well data with overlaid laboratory data.

The second effective attribute EA_2_ correlation with P- and S- wave impedance for well data is displayed in Fig. 8. The regression is developed on the base of well data; laboratory data is just overlaid on it to analyze global trend analysis. Except low impedance Monterey shale most of the points from are both datasets are following the same trend. Much better correlation can be seen for both P- and S-wave impedance. The square of linear regression coefficients R^2^ are 0.85 and 0.91 respectively. The liner equations obtained through this correlation are given below.

11a $${{\rm{EA}}}_{2}=30.5{I}_{P}-200.34$$

11b $${{\rm{EA}}}_{2}=45.6{I}_{S}-155.61$$

**Figure 9:**
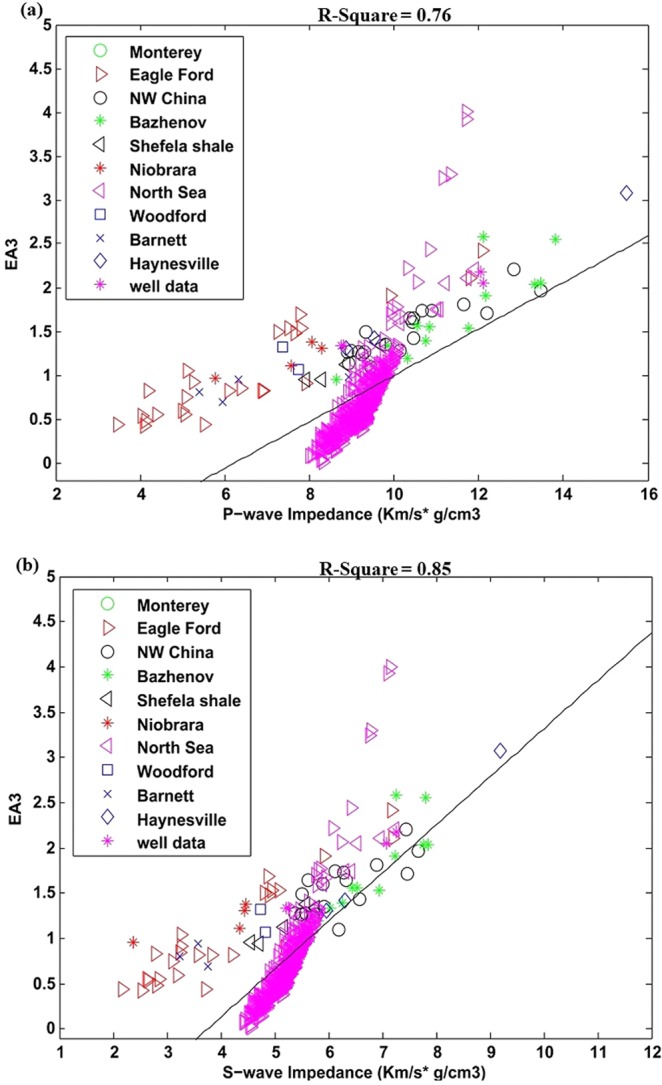
(**a**) EA_3_ correlation with P-wave impedance, for well data with overlaid laboratory data, (**b**) EA_3_ correlation with S-wave impedance for well data with overlaid laboratory data.

Finally, the effective attribute EA_3_ (brittleness index BI divided by sum of organic matter and Poisson’s ratio) for case of well data and its correlation P- and S- wave impedance is represented in Fig. 9. The laboratory data is also overlaid on it. The well data form Sembar shale has low brittleness index so both data are showing different trends. The square of linear regression coefficients is about 0.76 and 0.80. The equations obtained through linear correlation are given below.

12a $${{\rm{EA}}}_{3}=0.71{I}_{P}-5.92$$

12b $${{\rm{EA}}}_{3}=1.09{I}_{S}-5.0$$

### Seismic data application case study

**Figure 10:**
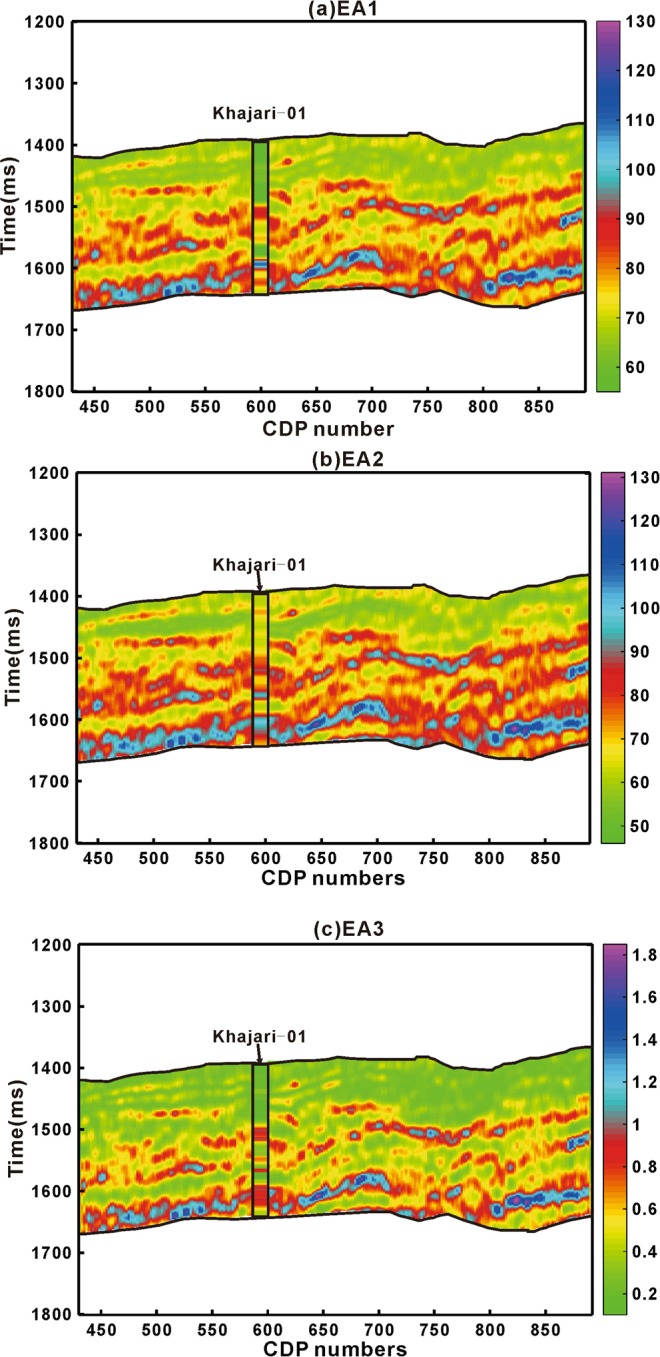
(**a**) EA_1_, (**b**) EA_2_ and (**c**) EA_3_ application on seismic data.

These effective attributes were applied on inverted 2D prestack P-wave impedance seismic data along well Khajari −01 whose target is organic shale reservoir. Across the time interval of desired zone inverted P- impedance section was transformed to EA_1_, EA_2_ and EA_3_ by using correlation obtained through Eqs. 10a,11a, and 12a (Fig. 10).

**Figure 11:**
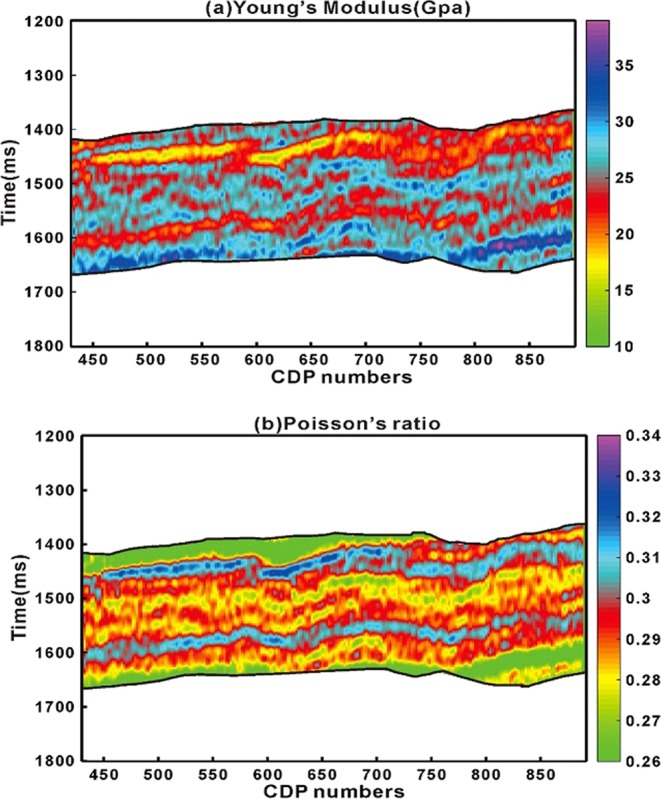
(**a**) Young’s Modulus and (**b**) Poisson’s ratio calculated from inverted seismic data.

As we defined EA_1_ in Eq. 3 which is combined effect of Young’s Modulus (E), Poisons ratio ($\nu$) and organic matter volume fraction. So, from inverted seismic data we have P- wave, S- wave velocity and density that can lead us to the direct measurement of Young’s Modulus and Poisons ratio (Fig. 11).

**Figure 12:**
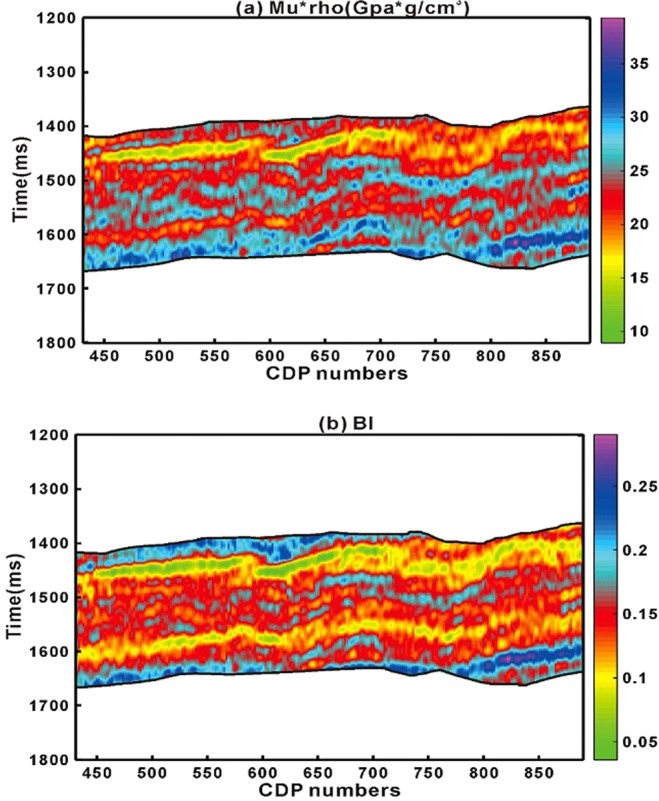
(**a**) Murho and (**b**) Elastic brittleness index calculated from inverted seismic data.

In EA_2_ for Eq. 4 and EA3 for Eq. 6 similarly the product of shear modulus and density (Murho) and elastic brittleness index (${BI}$) are directly derived from seismic data as shown in Fig. 12.

**Figure 13:**
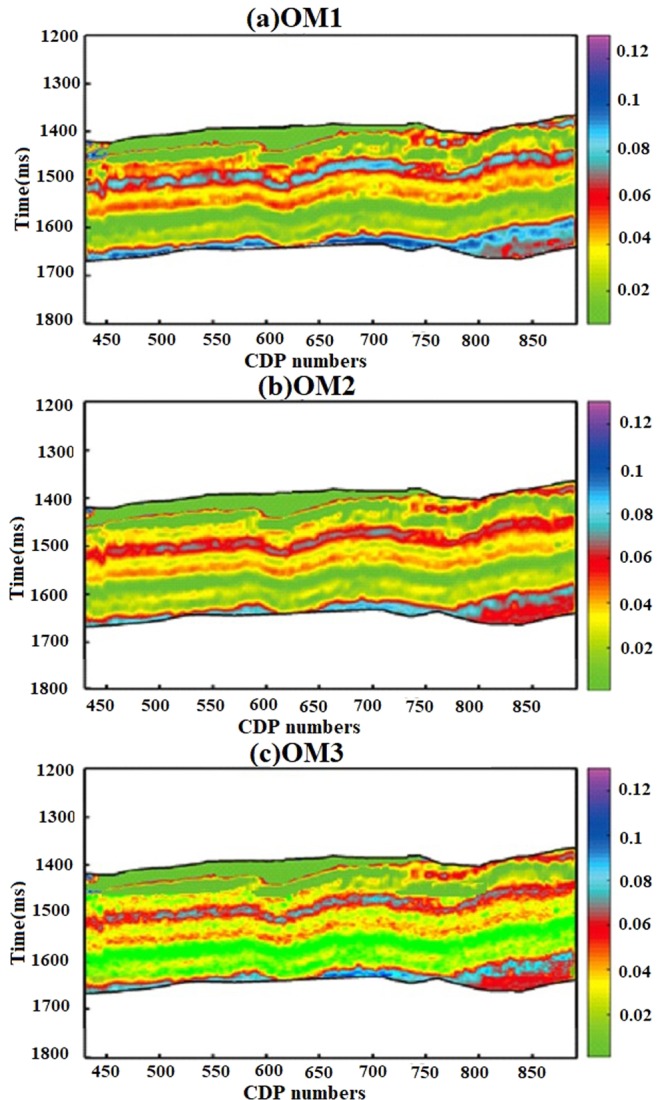
Organic matter (vol fraction) estimation across seismic scale by (**a**) EA_1_, (**b**) EA_2_ and (**c**) EA_3_.

The effect of all these Young’s Modulus (E), Poisons ratio (${\nu }$), Murho (${\mu }{\rho }$) and elastic Brittleness index (${BI}$) seismic derived sections of were omitted from original EA_1_, EA_2_ and EA_3_ sections which lead us to the rough estimation of organic matter across seismic scale (Fig. 13). The OM1, OM2 and Om3 are respectively attained by its respective attribute.

## Discussion

Due to existence of a gap between unconventional reservoir properties and seismic characteristics, it is challenge to directly to analyze these reservoir properties on seismic scale. We introduced effective attributes method that is based on the integration of different reservoir and elastic properties. This method is viable to reduces this gap and is helpful to analyze it on seismic data.

These all attributes can provide information about the shale brittleness and its hydrocarbon generation capacity. For effective attribute EA_1_, the Young’s modulus and Poisson’s ratio are the part of reservoir drilling while, organic matter is the part of hydrocarbon potential. This attribute has shown better correlation P-and S- wave impedance for the cases of both laboratory and well data (Figs. 3 and 7).

For second effective attribute EA_2_, Poisson’s ratio and shearing modulus can inform about geomechanical behaviors of the shales while organic matter can enlighten its capacity to generate hydrocarbon. This attribute has also shown successful result on both laboratory and well data (Figs. 4 and 8). The Well data is importantly exhibiting much better correlation for this reservoir attribute.

Elastic based brittleness index BI divided by sum of organic matter and Poisson’s ratio directly enlighten shale brittleness and its hydrocarbon generation capacity. Individual application of the attribute EA_3_ on laboratory and well data have shown suitable results while, in combined form both datasets are showing different trend (Figs. 5 and 9).

The effective attributes for well data with overlaid laboratory data helped us to analyze its global application trend. Most of the points in EA_1_ and EA_2_ for both laboratory and well data are following similar trend (Figs. 7 and 8). The effective attribute EA_3_ is showing different trend for both datasets (Fig. 9). One reason for this diverse trend is that there is large difference in brittleness index of Sembar shale (well data) with rest of laboratory data. The Laboratory datasets belongs to different regions, mineral contents, depths and environment of depositions that also affect correlation to certain degrees. The local application of this attribute can give better results.

It is shown that rock impedance is mainly controlled by both hard and soft components, rather than the single parameter. Brittleness index and different geomechanical properties are more sensitive to rock impedance as compared to organic matter. This is because these properties are directly derived from rock elastic properties (Fig. 1).

From seismic data generally we get combined response rather than response of individual reservoir properties. The simple linear regression model through proposed method on laboratory measured and well-logging data can be openly applied to seismic scale. From seismic case study example, we have shown that we can directly calculate geomechanical parameters for brittleness evaluation from inverted pre-stack seismic data rest of the part have led us to roughly estimate organic matter (Figs. 10,11,12 and 13). The accuracy is of each seismic derived parameter is largely dependent on available seismic data and its inversion results. If high frequency seismic data and more number of wells located on seismic line reliable results through proposed method can be achieved. Further the anisotropic effect on seismic data should also be taken into account.

Finally, it should also to be noticed that each shale reservoir is unique and heterogeneous in nature. This concept of hard and soft components response by brittleness and organic matter mainly applicable to reservoirs where both these components have contribution to rock elastic impedance. In some unconventional reservoir’s mineralogical composition particularly clay minerals or organic matter, porosity, pore fluids, pore structures, and pore pressure might be dominant to control elastic response. The relationship of organic matter and rock elastic response is quite complex because at certain level it also effects mechanical properties of the rock. For one reservoir organic matter itself might dominate the elastic properties of organic shale, and hence for the case P- or S-impedances might show better correlations individually as compared dividing rock into its hard and soft response. Depending on local rock conditions for example in specific shale reservoir one attribute may give better results while in other similar attribute may provide insufficient results. Due to all these specific concerns local calibration on well data and laboratory measurements must be analyzed to validate attributes application approach.

## Conclusions

With extensive analysis of worldwide laboratory dataset from different organic shale reservoirs we have found that by dividing unconventional reservoir properties into hard and soft components can get much improved linear correlation with P- and S- wave impedance.

The proposed three effective attributes help us to reduce the gap between organic shale reservoir properties and their seismic characteristics. These attributes simply enlighten the two most important aspects, hydrocarbon generation capacity and rock brittleness in unconventional reservoirs. The attributes application on well data example from Sembar shale have also shown successful results to link elastic properties with organic shale reservoir properties. The effective attributes EA_1_ and EA_2_ are showing same trend for both laboratory and well data which enlighten its global application.
